# Knockdown of RhoE Expression Enhances TGF-β-Induced EMT (epithelial-to-mesenchymal transition) in Cervical Cancer HeLa Cells

**DOI:** 10.3390/ijms20194697

**Published:** 2019-09-22

**Authors:** Makoto Nishizuka, Rina Komada, Masayoshi Imagawa

**Affiliations:** 1 Department of Applied Biology and Food Sciences, Faculty of Agriculture and Life Science, Hirosaki University, 3 Bunkyo-cho, Hirosaki, Aomori 036-8561, Japan; 2 Department of Molecular Biology, Graduate School of Pharmaceutical Sciences, Nagoya City University, 3-1 Tanabe-dori, Mizuho-ku, Nagoya, Aichi 467-8603, Japanimagawa@phar.nagoya-cu.ac.jp (M.I.)

**Keywords:** EMT (epithelial-to-mesenchymal transition), cervical cancer, RhoE, RhoA, TGF-β

## Abstract

Cervical cancer with early metastasis of the primary tumor is associated with poor prognosis and poor therapeutic outcomes. Since epithelial-to-mesenchymal transition (EMT) plays a role in acquisition of the ability to invade the pelvic lymph nodes and surrounding tissue, it is important to clarify the molecular mechanism underlying EMT in cervical cancer. RhoE, also known as Rnd3, is a member of the Rnd subfamily of Rho GTPases. While previous reports have suggested that RhoE may act as either a positive or a negative regulator of cancer metastasis and EMT, the role of RhoE during EMT in cervical cancer cells remains unclear. The present study revealed that RhoE expression was upregulated during transforming growth factor-β (TGF-β)-mediated EMT in human cervical cancer HeLa cells. Furthermore, reduced RhoE expression enhanced TGF-β-mediated EMT and migration of HeLa cells. In addition, we demonstrated that RhoE knockdown elevated RhoA activity and a ROCK inhibitor partially suppressed the acceleration of TGF-β-mediated EMT by RhoE knockdown. These results indicate that RhoE suppresses TGF-β-mediated EMT, partially via RhoA/ROCK signaling in cervical cancer HeLa cells.

## 1. Introduction

Cervical cancer is the third most common malignancy in women worldwide [1,2], and cervical cancer with early metastasis of the primary tumor is associated with poor prognosis and poor therapeutic outcomes [3,4]. Metastasis consists of sequential and selective steps, including local invasion, intravasation, circulation, extravasation, and colonization. Epithelial-to-mesenchymal transition (EMT) is an important step for intravasation of metastatic cancer cells into blood. During EMT, epithelial cells lose their polarity, cytoskeletal structure, and cell–cell adhesion and acquire migratory properties typical of mesenchymal cells [5,6,7]. It is thought that EMT plays a role in the acquisition of the ability to invade the pelvic lymph nodes and surrounding tissue in cervical cancer cells [1,2]. Therefore, it is important to clarify the molecular mechanisms underlying EMT in cervical cancer.

RhoE, also known as Rnd3, is a member of the Rnd subfamily of Rho GTPases. Unlike typical Rho proteins, RhoE is predominantly GTP-bound and is considered constitutively activated [8,9]. It is well known that RhoE regulates cytoskeletal reorganization and cell motility via inhibition of RhoA activity [10,11]. RhoE plays an important role in arresting cell cycle distribution, inhibiting cell growth, and inducing apoptosis and differentiation [8,11]. Furthermore, studies have shown that RhoE is also involved in cancer metastasis. For example, gain and loss-of-function experiments showed that RhoE increased the invasiveness and metastatic potential in several different cancer cell lines, including gastric cancer, prostate cancer, and melanoma [12,13,14]. On the other hand, Ma et al. showed that RhoE suppressed invasion of hepatocellular carcinoma [15]. In addition, ectopic RhoE expression reduced the invasive ability and metastasis of sarcoma cells [16].

RhoE may play a dual role in regulating EMT, depending on the cancer type. Zhou et al. reported that upregulation of RhoE expression by hypoxia promoted EMT in gastric cancer cells [17]. On the other hand, Grise et al. showed that attenuated RhoE expression in hepatocyte carcinoma led to downregulation of E-cadherin, an epithelial marker, and promoted EMT [18]. Therefore, RhoE may act as either a positive or negative regulator of cancer metastasis and EMT. However, the role of RhoE during EMT in cervical cancer cells remains unclear. In this study, we investigated the role and function of RhoE in transforming growth factor-β (TGF-β)-mediated EMT in HeLa cells. We found that knockdown of RhoE accelerated TGF-β -mediated EMT, partially via RhoA/ROCK signaling.

## 2. Results

### 2.1. RhoE Expression Is Elevated during TGF-β-Mediated EMT in HeLa Cells

**Figure 1:**
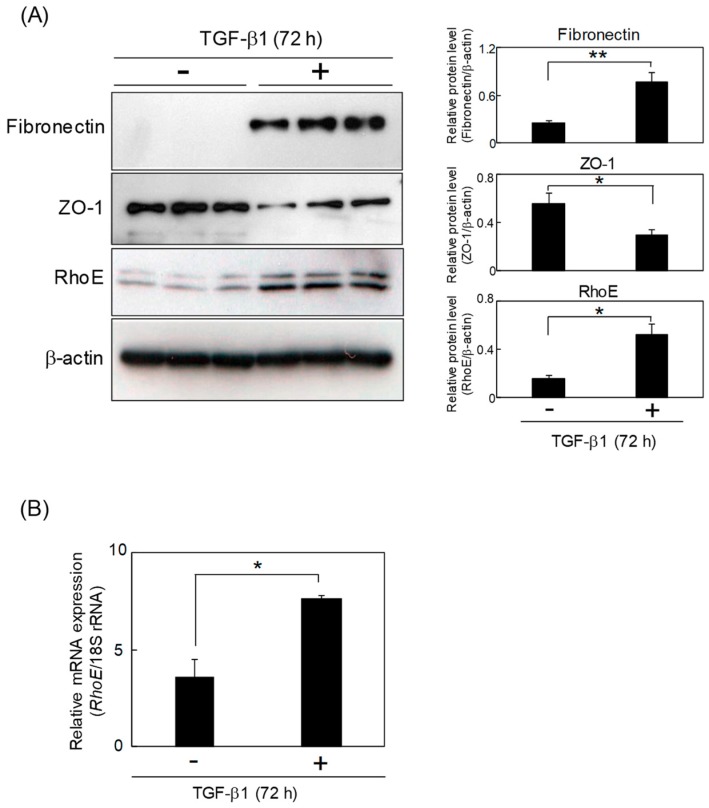
RhoE expression is elevated during transforming growth factor-β (TGF-β)-mediated epithelial-to-mesenchymal transition (EMT) in HeLa cells. (**A**) Protein levels of RhoE, fibronectin, and ZO-1 in HeLa cells after treatment with 1 ng/mL TGF-β1. Whole-cell lysates were subjected to Western blot analysis and β-actin was used as a loading control. The three lanes represent triplicates using cell lysates prepared from different culture plates. Signal intensities of the proteins were quantified using NIH-Image software. Data in each column represent the mean and standard deviation of three independent experiments. (**B**) qPCR analysis of RhoE expression in HeLa cells treated with TGF-β1. Expression levels of RhoE were normalized to 18S rRNA expression. Data in each column represent the mean and standard deviation of three independent experiments. The statistical significance of differences between two groups was evaluated using a two-tailed Student’s *t*-test. * *p* < 0.01 and ** *p* < 0.05.

We first examined RhoE expression levels during TGF-β-mediated EMT in HeLa cells. The expression of fibronectin, a mesenchymal marker, was upregulated by TGF-β treatment, whereas the expression level of ZO-1, an epithelial marker, was decreased. Western blot and quantitative real-time polymerase chain reaction (qPCR) analyses showed higher expression levels of RhoE in cells treated with TGF-β1 for 72 h compared with the control cells, suggesting that RhoE expression is elevated during TGF-β-induced EMT in HeLa cells (Figure 1).

### 2.2. RhoE Knockdown Enhances TGF-β-Mediated EMT in HeLa Cells

**Figure 2:**
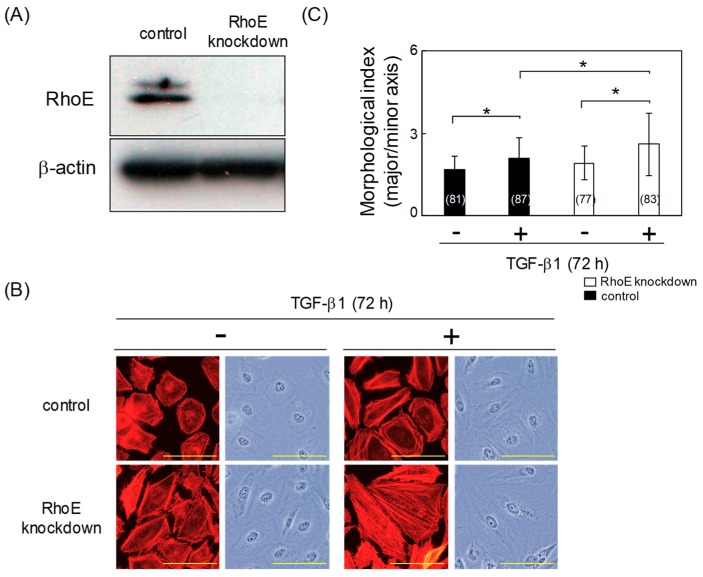
RhoE knockdown increases the degree of cell elongation in HeLa cells treated with TGF-β1. (**A**) Knockdown efficiency of RhoE in HeLa cells. HeLa cells were transfected with siRhoE-A. Luciferase small interfering RNA (siRNA) was used as a control and β-actin expression was used as a loading control. (**B**) Morphological changes in HeLa cells transfected with siRhoE-A. Cells were treated with 1 ng/mL TGF-β1 for 72 h. F-actin was visualized using tetramethylrhodamine isothiocyanate (TRITC)-conjugated phalloidin. Scale bars represent 100 μm. (**C**) Quantitative analysis of cell morphology of HeLa cells in (**B**). The lengths of the major and minor cell axes were measured using NIH-Image software. The ratios of the major to minor axes of cells were used to determine the degree of cell elongation. More than 77 cells were measured per condition for each experiment. Numbers used for the experiments are shown in parentheses. Data in each column represent the mean and standard deviation. Statistical significance was assessed using one-way ANOVA with a post hoc Tukey–Kramer HSD test. *p < 0.01. Similar results were obtained in two independent experiments.

To elucidate the role of RhoE in TGF-β-mediated EMT, HeLa cells were transfected with either a control small interfering RNA (siRNA) or an siRNA targeting RhoE (siRhoE-A). As shown in Figure 2A, transfection with siRhoE-A efficiently downregulated RhoE. F-actin was stained in RhoE knockdown and control cells using tetramethylrhodamine isothiocyanate (TRITC)-conjugated phalloidin. After TGF-β treatment, the number of long, thick actin stress fibers was increased in RhoE knockdown cells compared with control cells (Figure 2B). TGF-β significantly increased the degree of cell elongation in control and RhoE knockdown cells (Figure 2C). In addition, the degree of cell elongation was significantly increased in RhoE knockdown cells treated with TGF-β compared with that in control cells (Figure 2C).

**Figure 3:**
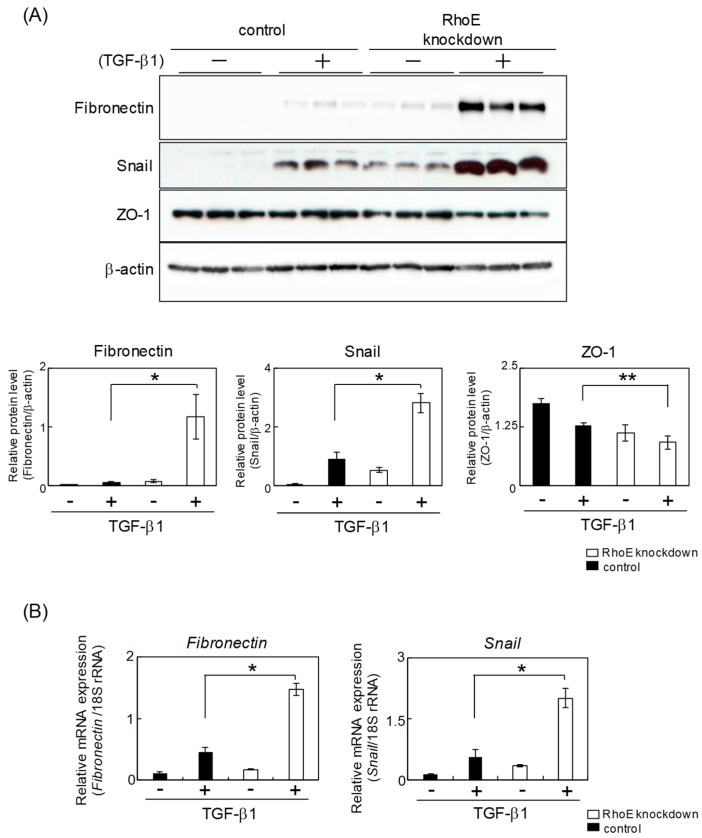
Knockdown of RhoE expression changes the expression levels of EMT-related genes in HeLa cells. (**A**) Protein expression of fibronectin, snail, and ZO-1 in HeLa cells transfected siRhoE-A. Cells were treated with 1 ng/mL TGF-β1 for 72 h. Whole-cell lysates were subjected to Western blot analysis and β-actin was used as a loading control. The three lanes represent triplicates using cell lysates prepared from different culture plates. Signal intensities of the proteins were quantified using NIH-Image software. Each column represents the mean and standard deviation of three independent experiments. (**B**) qPCR analysis of fibronectin and snail expression in RhoE knockdown cells. Expression levels of fibronectin and snail were normalized to 18S rRNA expression. Each column represents the mean and standard deviation of three independent experiments. Statistical significance was assessed using one-way ANOVA with a post hoc Tukey–Kramer HSD test. *p < 0.01 and **p < 0.05.

Next, we measured expression levels of EMT-related genes. Western blot analysis showed that protein levels of fibronectin and snail, a transcription factor promoting EMT, were significantly increased in RhoE knockdown cells, whereas expression levels of ZO-1 were decreased in RhoE knockdown cells treated with TGF-β compared with control cells (Figure 3A). mRNA expression levels of fibronectin and snail were also elevated in RhoE knockdown cells treated with TGF-β (Figure 3B). Knockdown experiments using RhoE-B, a siRNA-targeting region different from RhoE-A, also showed increased mesenchymal marker protein expression in addition to decreased epithelial marker protein in RhoE knockdown cells (Figure S1). These results suggest that a reduction of RhoE expression enhanced TGF-β-induced EMT in HeLa cells.

### 2.3. Reduction of RhoE Expression Enhances Migration of HeLa Cells during TGF-β-Mediated EMT

**Figure 4:**
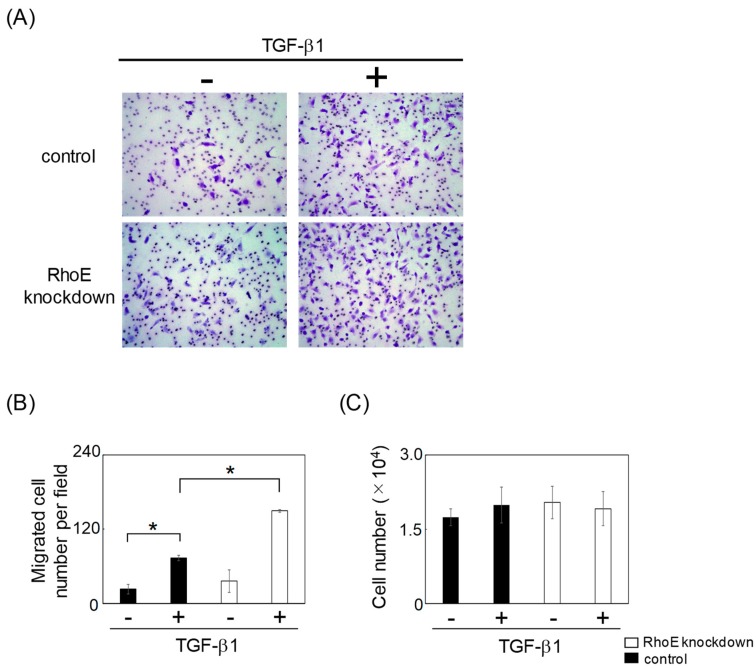
Knockdown of RhoE expression enhances the migration of HeLa cells undergoing TGF-β-mediated EMT. HeLa cells were transfected with siRhoE-A or a control siRNA and treated with 1 ng/mL TGF-β1 for 72 h. Cells undergoing EMT were plated in the upper chamber of the filters coated with fibronectin. Cells that migrated to the underside of the transwell insert were measured after 24 h. (**A**) Representative images of migrated cells are shown. (**B**) The mean number of migrated cells in the field was calculated. Data in each column represent the mean and standard deviation of three independent experiments. (**C**) Cells undergoing EMT were plated in culture plates. After 24 h, cells were trypsinized and counted. Data in each column represent the mean and standard deviation of three independent experiments. Statistical significance was assessed using one-way ANOVA with a post hoc Tukey–Kramer HSD test. *p < 0.01.

We next examined whether reduced RhoE expression levels influenced the migration ability of HeLa cells following TGF-β-mediated EMT. HeLa cells transfected with control or RhoE siRNA were treated with TGF-β1 for 72 h to induce EMT. Cells that had undergone EMT were seeded to fibronectin-coated transwell chambers. Knockdown of RhoE significantly increased the number of migrated cells (Figure 4A,B). On the other hand, RhoE knockdown had little influence on the growth of HeLa cells during EMT (Figure 4C). These results suggest that RhoE regulates the migration ability of HeLa cells that have undergone EMT.

### 2.4. RhoE Knockdown Enhances RhoA Activity during TGF-β-Mediated EMT in HeLa Cells

**Figure 5:**
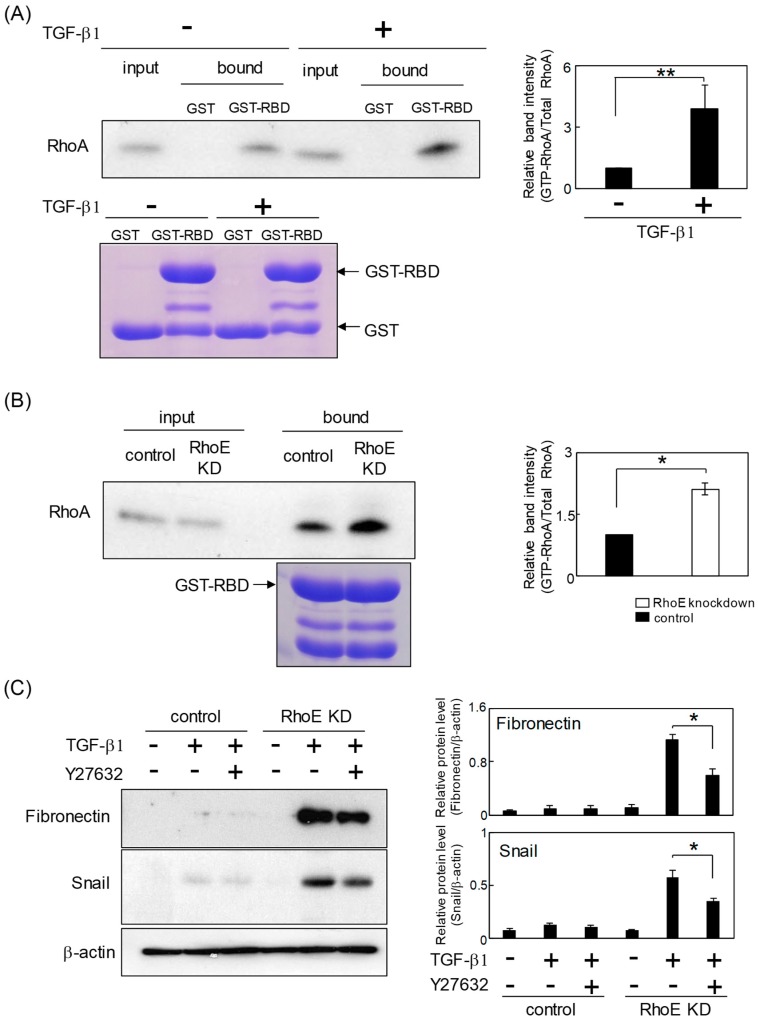
Reduction of RhoE upregulates RhoA activity during TGF-β-mediated EMT in HeLa cells. (**A**) Whole-cell lysates prepared from HeLa cells treated with TGF-β1 or vehicle were used for the GST pull-down assay with GST or GST–rhotekin Rho binding domain (RBD). The lower panel indicates Coomassie blue staining of GST proteins. Bound protein samples were immunoblotted with anti-RhoA antibody. The input volume was 5% of that of the cell lysates for the pull-down assay. The ratio of GTP-RhoA/total RhoA was quantified. The immunoblot shows the representative result and data in each column represent the mean and standard deviation of three independent experiments. The statistical significance of differences between two groups was evaluated using a two-tailed Student’s *t*-test. ** *p* < 0.05. (**B**) HeLa cells were transfected with siRhoE-A or a control siRNA and treated with 1 ng/mL TGF-β1 for 72 h. Whole-cell lysates were used for the GST pull-down assay with GST–rhotekin RBD. Bound protein samples were immunoblotted using anti-RhoA antibody. The input volume was 5% of that of the cell lysates for the pull-down assay. The ratio of GTP-RhoA/total RhoA was quantified. The immunoblot shows the representative result and data in each column represent the mean and standard deviation of three independent experiments. The statistical significance of differences between two groups was evaluated using a two-tailed Student’s *t*-test. * *p* < 0.01. (**C**) Protein expression of fibronectin and snail in control and RhoE knockdown cells treated with Y27632. HeLa cells were transfected with siRhoE-A or a control siRNA and pretreated with or without 10 μM Y27632 for 2 h followed by stimulation with vehicle or TGF-β1 for 72 h. Whole-cell lysates were subjected to Western blot analysis and β-actin was used as a loading control. Signal intensities of proteins were quantified using NIH-Image software. Immunoblots show representative results and each column represents the mean and standard deviation of three independent experiments. Statistical significance was assessed using one-way ANOVA with a post hoc Tukey–Kramer HSD test. * *p* < 0.01 and ** *p* < 0.05.

TGF-β activates RhoA to induce stress fiber formation and mesenchymal characteristics [19,20]. A rhotekin assay was used to test whether RhoA activity was upregulated during TGF-β-mediated EMT in HeLa cells. Treatment with TGF-β led to an increased amount of GTP-bound RhoA protein, indicating that TGF-β activates RhoA in HeLa cells (Figure 5A). We next examined the effects of RhoE knockdown on RhoA activity during TGF-β-induced EMT. As shown in Figure 5B, the ratio of GTP-RhoA/total RhoA was significantly increased in RhoE knockdown cells compared with that in control cells. These results suggest that reduced RhoE expression enhances RhoA activity during TGF-β-induced EMT in HeLa cells. Finally, we examined whether RhoE regulates TGF-β-mediated EMT via RhoA/ROCK signaling. As shown in Figure 5C, pretreatment with Y27632, a selective ROCK inhibitor, partially, but significantly, suppressed the elevated expression of mesenchymal markers in RhoE knockdown cells treated with TGF-β. This result suggests that RhoA/ROCK signaling contributes to the promotion of TGF-β-mediated EMT by RhoE knockdown.

## 3. Discussion

Cancer metastasis accounts for the majority of cancer deaths [21,22]. In cervical cancer, pelvic lymph node metastasis is the major route for tumor metastasis and is an important risk factor for recurrence or death [3,4]. TGF-β is a multifunctional cytokine that is associated with cancer metastasis and promotes EMT in various cancer cells [23,24]. While it is well known that TGF-β-induced EMT is regulated by many factors, such as Smad2/3, Snail, Slug, and Twist [23,24,25,26], the underlying mechanisms involved in TGF-β-mediated EMT in cervical cancer are not fully understood. The present study showed that reduced RhoE expression enhanced TGF-β-mediated EMT, suggesting that RhoE functions as a negative regulator of TGF-β-induced EMT in cervical cancer cells.

RhoA is member of the Ras superfamily of small GTPases, and its activation induces the assembly of stress fibers and cytoskeleton-mediated changes in cell motility [27,28]. Studies have shown that TGF-β promotes cell invasion and EMT via activation of RhoA. For example, Bhowmick et al. reported that blocking RhoA activity inhibited TGF-β-mediated EMT in epithelial cells [19]. Korol et al. showed that TGF-β-induced cytoskeletal reorganization via RhoA/ROCK signaling was critical for EMT in lens epithelial cells [29]. Furthermore, farnesyl pyrophosphate synthase promoted TGF-β-induced EMT via RhoA activation in non-small-cell lung cancer cells [30]. Since it is established that RhoA is associated with cervical cancer metastasis [31,32], the TGF-β-RhoA signaling pathway must also play an important role in the regulation of metastasis of cervical cancer cells.

In the present study, knockdown of RhoE expression enhanced RhoA activity, implying that acceleration of TGF-β-mediated EMT in RhoE knockdown cells is accompanied by increased RhoA activation. There are two possible mechanisms by which RhoE may control the activity of RhoA signaling. One is that RhoE may bind directly to ROCK1, a RhoA target effector, and inhibit its activity [33]. The other is that RhoE may interact with p190RhoGAP, a negative RhoA regulator, to reduce cellular levels of RhoA–GTP [34]. It is reported that both ROCK1 and p190RhoGAP contribute to the promotion of cancer metastasis and EMT in various cancer cells [35,36]. As shown in Figure 5C, the use of a ROCK inhibitor demonstrated that RhoA/ROCK signaling plays an important role in the acceleration of TGF-β-mediated EMT by RhoE knockdown. On the other hand, there was only a partial suppression of the expression of mesenchymal markers, suggesting that RhoE may also regulate TGF-β-induced EMT through pathways other than RhoA/ROCK signaling. It is reported that RhoA activates several effector proteins, such as phosphotidylinositide 4P-5 kinase and mDia, in addition to ROCK [37,38]. Furthermore, TGF-β induces a multitude of signal transducers, including Smad2/3, mitogen-activated protein kinases, phosphoinositide-3-kinase, and Akt [39,40]. It is necessary to examine whether RhoE regulates these factors during TGF-β-mediated EMT in HeLa cells. Monaghan-Benson et al. demonstrated that knockdown of RhoE caused a decrease in p190GAP activity, an increase in RhoA activity, and an increase in the expression of fibronectin in normal lung fibroblasts [34]. Furthermore, they showed that TGF-β treatment decreased p190GAP activity and increased RhoA activity [34]. It is necessary to examine the role of the RhoE/p190 pathway in TGF-β-mediated EMT in cervical cancer cells.

Although a significant difference was not observed, RhoE knockdown slightly facilitated the migration of HeLa cells that had not undergone TGF-β-mediated EMT (Figure 4). Since previous reports demonstrated that RhoE suppressed the TGF-β-independent migration of several cancer cells, including hepatocellular carcinoma [15,16], RhoE may also regulate TGF-β-independent cell migration in HeLa cells.

As shown in Figure 1, expression levels of RhoE were elevated in HeLa cells treated with TGF-β. Furthermore, knockdown experiments showed that RhoE negatively regulated TGF-β-induced EMT in HeLa cells (Figure 2 and Figure 3). These findings suggest that RhoE acts as a regulator of TGF-β signaling via negative feedback loops as well as Smad7 and SnoN [41,42]. Since RhoE lacks intrinsic GTPase activity and remains in its active GTP-bound form, it is important to elucidate the mechanism of regulation of RhoE expression. Zhou et al. showed that RhoE expression was transcriptionally induced by hypoxia via hypoxia-inducible factor-1α in gastric cancer [17]. Furthermore, RhoE was shown to be upregulated by cyclic AMP via activation of protein kinase A in human BeWo choriocarcinoma cells [43]. In addition, several microRNAs, including miR-200b, miR-200c, and miR-17, regulate RhoE expression in various cancer cells [44,45,46]. Taken together, these findings indicate complex control of RhoE expression levels. It is also reported that RhoE has a diverse expression pattern depending on the cancer type [47]. Furthermore, a previous report showed that TGF-β downregulates the expression level of RhoE in normal lung fibroblasts [34]. Further analyses, including promoter analysis, are required to clarify how TGF-β regulates RhoE expression during EMT in cervical cancer.

In summary, knockdown experiments suggested that RhoE functions as a negative regulator of TGF-β-induced EMT in HeLa cells. Furthermore, we showed that RhoA/ROCK signaling partially contributes to the acceleration of TGF-β-mediated EMT by RhoE knockdown. Further functional studies of RhoE should throw light on the molecular mechanism of EMT and metastasis in cervical cancer.

## 4. Materials and Methods

### 4.1. Cell Culture

HeLa cells were purchased from RIKEN Cell Bank and cultured in minimum essential medium (MEM) containing 10% calf serum. Recombinant human TGF-β1 was purchased from R&D Systems (Minneapolis, MN, USA).

### 4.2. RNA Interference Experiments

Human RhoE siRNA and luciferase siRNA were purchased from Nippon EGT (Toyama, Japan) and transfected into HeLa cells using Lipofectamine2000 as previously described [48]. The sequences of siRhoE-A and siRhoE-B were 5′-CUGCCAGUUUUGAAAUCGA-3′ and 5′-GCAGCUACUUAUAUCGAAU-3′, respectively. Luciferase siRNA, 5′-CGUACGCGGAAUACUUCGATT-3′, was used as a control.

### 4.3. F-Actin Staining and Quantification of Elongated Cell Morphology

HeLa cells transfected with control siRNA or siRhoE were treated with TGF-β1 for 72 h. After washing, cells were fixed for 10 min in 2% paraformaldehyde and stained with TRITC-conjugated phalloidin to detect F-actin. Measurements of elongated cell morphology were performed as previously described [48]. The lengths of the major and minor cell axes were measured using NIH-Image software (https://imagej.nih.gov/ij/) and the ratios of the major to minor axes of cells were used to determine the degree of elongated cell morphology. More than 77 cells were measured per condition for each experiment.

### 4.4. Quantitative Real-Time PCR

Total RNA was extracted using RNAiso Plus (TaKaRa, Siga, Japan) according to the manufacturer’s instructions. Reverse transcription and qPCR were performed as previously described [49]. The specific primers for human RhoE, snail, fibronectin, and 18S rRNA were as follows: human RhoE-forward, 5′-AATAGAGTTGAGCCTGTGGG-3′; human RhoE-reverse, 5′-CTAATGTACTAACATCTGTCCGC-3′; human snail-forward, 5′-CCTCAAGATGCACATCCGAAG-3′; human snail-reverse, 5′-ACATGGCCTTGTAGCAGCCA-3′; human fibronectin-forward, 5′-GTGTTGGGAATGGTCGTGGGGAATG-3′; human fibronectin-reverse, 5′-CCAATGCCACGGCCATAGCAGTAGC-3′; human 18S rRNA-forward, 5′-CTCAACACGGGAAACCTCAC-3′; and human 18S rRNA-reverse, 5′-AGACAAATCGCTCCACCAAC-3′.

### 4.5. Western Blotting

Cells were lysed and equal amounts of total protein were resolved using sodium dodecyl sulfate (SDS)-polyacrylamide gel electrophoresis, then transferred to a polyvinylidene difluoride membrane and probed using primary antibodies and secondary antibodies conjugated with horseradish peroxidase (Jackson ImmunoResearch Laboratories, West Grove, PA, USA). Specific proteins were detected using an enhanced chemiluminescence system (GE Healthcare, Little Chalfont, UK). Primary antibodies against RhoE, fibronectin (Santa Cruz Biotechnology, Dallas, TX, USA), Snail, ZO-1 (Cell Signaling Technology, Danvers, MA, USA), and β-actin (SIGMA, St. Louis, MO, USA) were used. Quantification of the band intensity of the blots was performed using NIH-Image software.

### 4.6. Migration Assays

Migration assays using transwell plates were performed as previously reported with slight modifications [48]. After incubation with TGF-β1 for 72 h, HeLa cells transfected with either control siRNA or siRhoE were trypsinized and transferred to fibronectin-coated inserts in serum-free medium. The cells were then incubated in MEM supplemented with 10% calf serum for 24 h. Cells on the upper surface of the membrane were removed by scrubbing with cotton swabs. Chambers were fixed in 4% paraformaldehyde for 10 min and stained with crystal violet. Cells that penetrated the filter were observed with a microscope and counted.

### 4.7. Rhotekin Assays

RhoA activity was determined by binding to GST–rhotekin– Rho binding domain (RBD). HeLa cells transfected with control siRNA or siRhoE were treated with TGF-β1 for 72 h then lysed. Cell lysates were incubated for 24 h with GST–rhotekin–RBD on glutathione–sepharose beads. The beads were resuspended with 2× SDS sample buffer and boiled for 5 min. Protein samples were analyzed by Western blotting using anti-RhoA antibody.

### 4.8. Statistical Tests

Analyses were performed using Excel 2010 and R (http://cran.r-project.org/). The statistical significance of differences between two groups was evaluated using a two-tailed Student’s *t*-test. For multigroup analysis, significance was assessed using one-way ANOVA with a post hoc Tukey–Kramer HSD test. 

## Supplementary Materials

Supplementary materials can be found at https://www.mdpi.com/1422-0067/20/19/4697/s1.

## Abbreviations

EMT	Epithelial-to-mesenchymal transition
TGF-β	Transforming growth factor-β
TRITC	Tetramethylrhodamine isothiocyanate
RBD	Rho binding domain
