# Supplementary material for: Knockdown of RhoE Expression Enhances TGF-β-Induced EMT (epithelial-to-mesenchymal transition) in Cervical Cancer HeLa Cells
Source: Int J Mol Sci. 2019 Sep 22;20(19):4697. doi: 10.3390/ijms20194697 (PMC6801947; doi:10.3390/ijms20194697)
Supplement: Supplementary file 1 [file ijms-20-04697-s001.pdf]

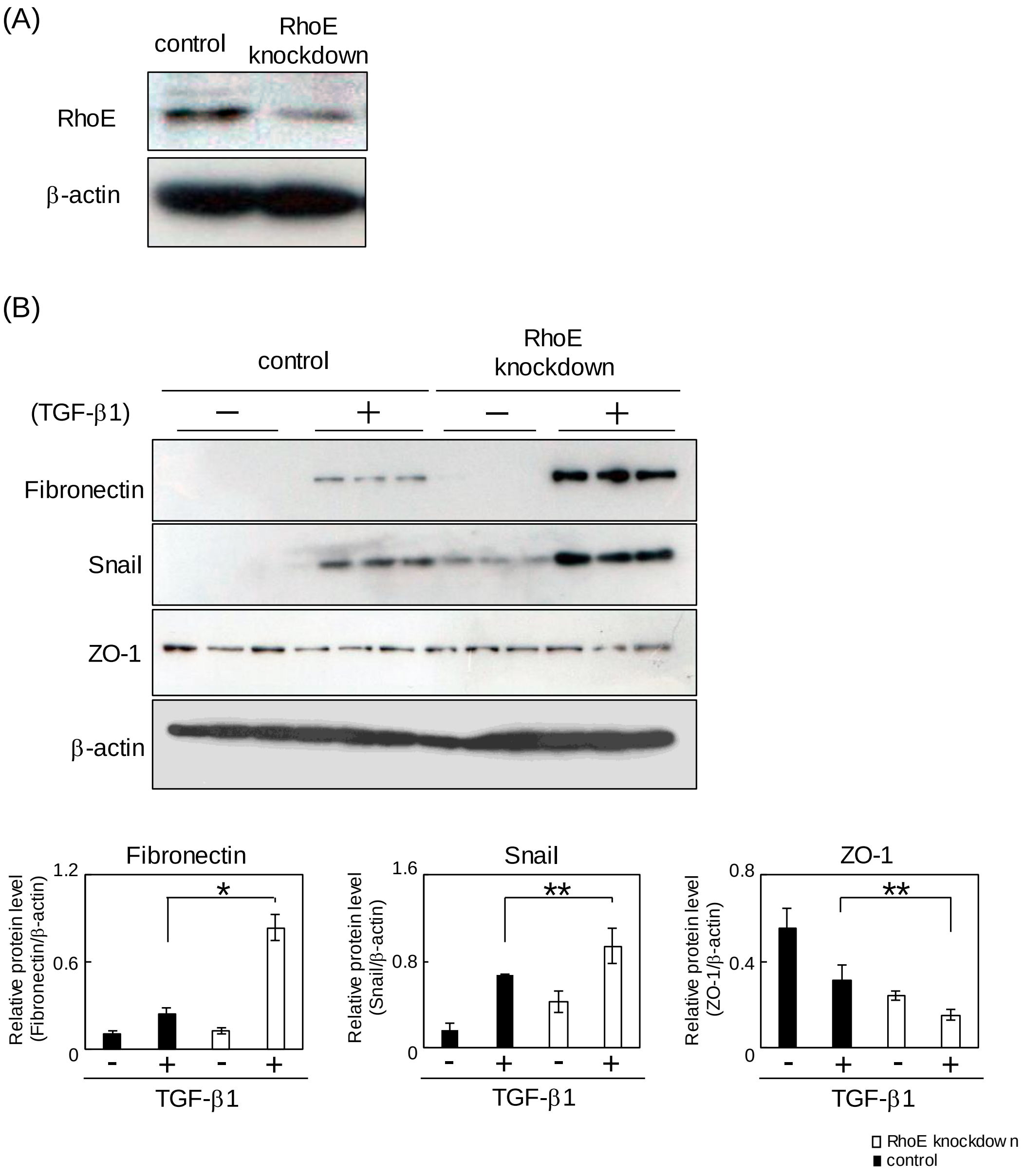

Supplementary Figure 1 Nishizuka *et al.*

Supplementary Figure 1. RhoE knockdown using siRhoE-B changes the expression levels of EMT-related genes in HeLa cells. (A) Knockdown efficiency of RhoE in HeLa cells. HeLa cells were transfected with siRhoE-B. Luciferase siRNA was used as a control and β-actin expression was used as a loading control. (B) Protein expression of fibronectin, Snail, and ZO-1 in HeLa cells transfected siRhoE-B. Cells were treated with 1 ng/mL TGF-β1 for 72 h. Whole-cell lysates were subjected to Western blot analysis and β-actin was used as a loading control. The three lanes represent triplicates using cell lysates prepared from different culture plates. Signal intensities of the proteins were quantified using NIH-Image software. Each column represents the mean and standard deviation of three independent experiments. Statistical significance was assessed using one-way ANOVA with post hoc Tukey–Kramer HSD test. $^{*}p < 0.01$ and $^{**}p < 0.05$.
